# Excitatory-Inhibitory Homeostasis and Diaschisis: Tying the Local and Global Scales in the Post-stroke Cortex

**DOI:** 10.3389/fnsys.2021.806544

**Published:** 2022-01-10

**Authors:** Francisco Páscoa dos Santos, Paul F. M. J. Verschure

**Affiliations:** ^1^Eodyne Systems SL, Barcelona, Spain; ^2^Laboratory of Synthetic, Perceptive, Emotive and Cognitive Systems (SPECS), Institute for Bioengineering of Catalonia (IBEC), Barcelona, Spain; ^3^Department of Information and Communications Technologies (DTIC), Universitat Pompeu Fabra (UPF), Barcelona, Spain; ^4^Catalan Institution for Research and Advanced Studies (ICREA), Barcelona, Spain

**Keywords:** stroke, diaschisis, cortical reorganization, functional networks, excitability, homeostatic plasticity, Excitatory-inhibitory balance

## Abstract

Maintaining a balance between excitatory and inhibitory activity is an essential feature of neural networks of the neocortex. In the face of perturbations in the levels of excitation to cortical neurons, synapses adjust to maintain excitatory-inhibitory (EI) balance. In this review, we summarize research on this EI homeostasis in the neocortex, using stroke as our case study, and in particular the loss of excitation to distant cortical regions after focal lesions. Widespread changes following a localized lesion, a phenomenon known as diaschisis, are not only related to excitability, but also observed with respect to functional connectivity. Here, we highlight the main findings regarding the evolution of excitability and functional cortical networks during the process of post-stroke recovery, and how both are related to functional recovery. We show that cortical reorganization at a global scale can be explained from the perspective of EI homeostasis. Indeed, recovery of functional networks is paralleled by increases in excitability across the cortex. These adaptive changes likely result from plasticity mechanisms such as synaptic scaling and are linked to EI homeostasis, providing a possible target for future therapeutic strategies in the process of rehabilitation. In addition, we address the difficulty of simultaneously studying these multiscale processes by presenting recent advances in large-scale modeling of the human cortex in the contexts of stroke and EI homeostasis, suggesting computational modeling as a powerful tool to tie the meso- and macro-scale processes of recovery in stroke patients.

## Stroke and Diaschisis

In stroke, disruptions in blood flow in the central nervous system lead to focal lesions in the brain or spinal cord, causing it to be one of the most burdening disorders in economically advantaged countries (Campbell and Khatri, 2020). As a result of such lesions, patients experience a broad range of symptoms, with deficits in motor (e.g., hemiparesis), sensory (e.g., hemianopia) and higher-order cognitive processes (e.g., aphasia, hemispatial neglect) (Musuka et al., 2015; Campbell and Khatri, 2020), even leading to neuropsychiatric deficits such as depression (Towfighi et al., 2017) and dementia (Leys et al., 2005). While some of the effects of stroke can be directly attributed to loss of function of lesioned areas (Siegel et al., 2016), its effects extend beyond the lesioned area involving multiple areas across the cortex, a phenomenon known as diaschisis. The term diaschisis, coined by von Monakow (1914), first pertained to a remote loss in excitability following focal lesion impacting the function of brain areas distant to the lesion. Since, then the topic of diaschisis was further elaborated in the following century (Feeney and Baron, 1986), mainly focusing on changes in excitability affecting the local excitatory-inhibitory (EI) balance of distant cortical networks. However, the measurable effects of stroke are not limited to the mesoscale of disruptions in EI balance, extending into large-scale cortical dynamics, such as functional interactions between distant regions. Therefore, extensions to the concept of diaschisis have been proposed in recent years, suggesting remote disruptions in functional connectivity as a relevant aspect of the process (Campo et al., 2012; Carrera and Tononi, 2014). With this recent expansion, attempting to bridge these two types of diaschisis emerging on different spatial scales is, therefore, a relevant issue, not only to better understand possible common physiological causes, but also to inform therapeutical strategies, thus improving post-stroke recovery. That said, in this review, we summarize the main findings related to diaschisis, both regarding functional connectivity (FC) and excitability, and link long-term changes in excitability to cortical plasticity mechanisms related to EI homeostasis, suggesting that the effects of these local processes extend beyond the scale of local EI balance regulation, into large-scale network dynamics. In addition, we summarize the recent advances in computational modeling of stroke, proposing modeling as a framework for the study of the concurrent evolution of FC and excitability in the post-stroke brain.

## Diaschisis and Functional Connectivity

A number of studies have elaborated on remote changes in functional connectivity, either at rest or task-related, following a stroke. FC quantifies the interaction or coupling between two chosen areas in the brain and is usually computed as the temporal correlation between their neural activity, although different methods (e.g., spectral coherence, transfer entropy) can be used depending on the nature of the signals (Friston, 2011). While studies on task-related FC have been performed on stroke patients (Gerloff et al., 2006; Westlake and Nagarajan, 2011), the use of resting state FC has some advantages, even though it does not provide specific information about the connectivity and activation of particular networks in the brain when subjects are engaged in behaviors of interest (Carter et al., 2012). Shortly, resting-state protocols have no requirements regarding patient function, thus allowing network analysis of patients with stronger impairments. Also, due to their unspecific nature, they allow a more global analysis of changes in connectivity, without the need for *a priori* assumptions about regions of interest. With that in mind, this section will focus on studies regarding resting-state FC changes in the post-stroke brain and how they relate to recovery (Carter et al., 2012).

**FIGURE 1:**
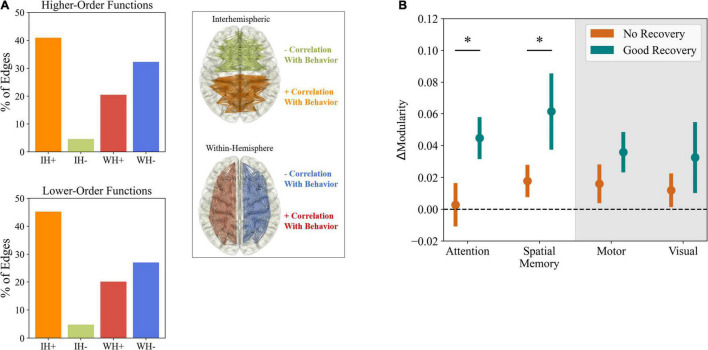
Post-stroke changes in functional connectivity. **(A)** Predictive value of disruptions in functional connections for different behavioral deficits. The bar charts represent the percentage of connections, from the top 200 predicting behavior, belonging to each of the four groups described in the right (e.g., a percentage of 30% of IH+ means that increases in interhemispheric connectivity represent 30% of the 200 FC changes that best predict behavior). Notably, the strongest predictors of performance were increased interhemispheric connectivity and decreased intrahemispheric connectivity, both for higher (e.g., attention and working memory) and lower-order functions (e.g., motor and visual). Adapted from Siegel et al. (2016). **(B)** Correlation between changes in modularity and functional recovery. Patients with good behavioral recovery in higher-order functions, from attention to language, showed significantly higher changes in modularity than patients with poor recovery. The same pattern was not observed for motor and visual functions. ΔModularity represents the slope of a logarithmic fit to modularity at 2 weeks, 3 months, and 1 year. Adapted from Siegel et al. (2018). * Represents *p* < 0.05, from one-tailed unpaired *t*-test, after correction for multiple comparisons.

Some of the first studies regarding changes in FC after stroke focused on changes in the connectivity strength between particular brain regions. Initial studies (He et al., 2007) showed an acute disruption in interhemispheric connectivity of the posterior intraparietal sulcus, part of the dorsal attention network, in patients with structural lesions involving the ventral attention network. The deficit correlated with spatial neglect and was normalized after 6 months, fitting the requirements of diaschisis (Carrera and Tononi, 2014). These findings were further confirmed in patients with an array of lesions in different regions (Carter et al., 2009; Baldassarre et al., 2014), showing an additional correlation between disrupted interhemispheric connectivity of the somatomotor network and motor. The authors emphasize these decreases in interhemispheric connectivity as predictive of behavioral deficit, as opposed to changes in intrahemispheric connectivity, which were less related to patient performance. However, a later study on post-stroke changes in the connectivity of the motor cortex (Park et al., 2011) showed that behavioral deficits could be explained not only by decreased interhemispheric connectivity but also by increases in its intrahemispheric counterpart. While research has focused on the motor and, to some extent, attention networks, changes are not confined to those particular systems. Indeed, it has been shown that, even in subcortical stroke patients, changes in connectivity are experienced at a brain-wide level, with a generalized increase in internal FC in a range of areas, from the visual to the dorsal attention networks (Wang et al., 2014). At the same time, decreases were found more particularly in the anterior default-mode and ventral attention networks. In this case, since measurements were taken more than 6 months after stroke onset, it is likely that these changes were not related to a direct effect of focal lesions, but instead the consequence of cortical reorganization. When looking at global metrics of functional connectivity and its changes, the biomarkers of inter and intrahemispheric connectivity are evident again. In the longitudinal study of Siegel et al. (2016), it becomes clear again that a general decrease in interhemispheric connectivity is observed during the subacute period in stroke patients, being gradually corrected across time. Importantly, a similar but opposite variation was detected for intrahemispheric connectivity, constituting, together with decreased interhemispheric connectivity, a strong biomarker of impairment (Figure 1A). Not only that, but the magnitudes of both changes correlated with each other, showing that they co-evolve and might be equally important in the process of recovery. While the authors indicate that visual and motor impairments were better predicted by lesion site, the two biomarkers provided a strong predictor of the recovery of higher-order functions, such as memory and language, which is not unexpected since these more integrative functions are thought to engage larger and more distributed networks (Goldman-Rakic, 1988; Mesulam, 1990). In a further paper regarding the low dimensionality of behavioral deficits in stroke patients, the authors hypothesized a matching low dimensionality in FC abnormalities, considering the two biomarkers of inter and intrahemispheric connectivity to be the strongest correlates of stroke-related deficits (Corbetta et al., 2018). However, as in less global metrics of functional connectivity, further research casts doubt into the role of intrahemispheric connectivity in stroke. A study using magneto encephalography (MEG) derived alpha-band connectivity (Westlake et al., 2012) showed increased intrahemispheric connectivity in the lesioned hemisphere, which was associated with better motor recovery, thus contradicting previous results (Siegel et al., 2016). Nonetheless, connectivity in the alpha band (8–12 Hz) may be reflective of different neurological processes that functional magnetic resonance imaging (fMRI) resting-state FC, which instead relies on much slower signals, in the order of 0.1 Hz (Cabral et al., 2011; Magri et al., 2012).

The study of post-stroke FC is, however, not limited to changes in the magnitude of connectivity between regions, and several examples can be found where more complex graph properties of functional networks have been related to stroke deficits and progression. One of the first examples is the work of Achard et al. (2006). By looking at the effects of progressive removal of nodes in an fMRI-derived FC graph, they showed that human functional networks were characterized by a small-world topology (Bassett and Bullmore, 2006) and were more resilient to progressive node removal than equivalent random and scale-free networks regarding the preservation of graph properties. While not particularly targeted at stroke, the work of Achard et al. (2006) already hinted at some of the later measured effects of local lesions in the graph properties of human FC. Indeed, in a subsequent study (Sporns et al., 2007), by analyzing data from cat and macaque cortical networks, it was found that removing structural connector hubs (nodes that connect different modules in the cortical network) leads to an increase in the small-world index of functional networks. This was explained by an increased path-length and clustering coefficient, indicative of segregation between areas in the analyzed cortical networks (Rubinov and Sporns, 2010). Further research has then confirmed the stronger effects of lesioning connector hubs, as opposed to lesions in sub-network hubs, by measuring the integrity of functional network organization (Gratton et al., 2012). One of the most common effects of stroke in human functional networks is an increase in segregation between areas in the brain (Sporns et al., 2007). This effect is evident not only by changes in metrics of segregation such as clustering coefficients, but also in measures of integration, as quantified by decreased local (Philips et al., 2017) and global (Philips et al., 2017; Zhang et al., 2017) efficiency or increased shorter path lengths (Zhang et al., 2017) in stroke patients. Interestingly, it might be the case that the effects on the graph properties of FC are more complex and involve changes in segregation and integration that are network specific. In that regard, measurements of modularity provide useful insights. Modularity measures the balance between the degree of integration within and segregation between functional networks (Siegel et al., 2016). In a longitudinal study of stroke patients (Siegel et al., 2018), it was found that modularity was significantly reduced in the acute period, being partially recovered after 3 and 12 months. As in previous results (Siegel et al., 2016), these FC changes were more correlated to higher-order functions such as memory and language, pointing toward the importance of global network integrity for their performance and justifying a particular vulnerability of more integrated functions to widespread damage, as opposed to sensory functions which engage more peripheral areas of cortical functional networks (Figure 1B). Further studies expanded on the use of modularity as a post-stroke biomarker and researchers were able to explain its reduction by connecting it to lesions in connector hubs, as opposed to provincial hubs (Gratton et al., 2012) and, more recently, to structural disconnection, representing damage to white matter tracts connecting regions (Griffis et al., 2019). That considered, the evidence seems to indicate that decreasing segregation in functional networks to pre-lesion levels would be beneficial for patient recovery. Indeed, Wang et al. (2010), when looking at longitudinal fMRI from the motor execution network of stroke patients, found a negative correlation between clustering coefficients and recovery of motor function. In addition, a positive correlation with recovery was found for betweenness centrality, which quantifies the importance of individual nodes in the flow of information in the network.

While most of the discussed changes pertain to cortico-cortical networks, given the scope of this review, the effects of stroke in functional connectivity do extend to the realm of cortico-subcortical interactions. An example of such is the aforementioned effect of subcortical lesions, which have been shown to impact cortical connectivity in a widespread manner (Wang et al., 2010, 2014; Siegel et al., 2016; Zhang et al., 2017), although not as strongly as cortical lesions (Philips et al., 2017). Another example is the effect of stroke in cortico-subcortical connectivity, leading to longitudinal changes in connectivity between cerebellum, thalamus and motor areas (Wang et al., 2010), which were found to correlate with motor recovery (Park et al., 2011). That said, although cortico-subcortical interactions might be of relevance, since a significant number of lesions are experienced in subcortical regions (Corbetta et al., 2018), we focus on cortical networks, not only because they are equally impacted by subcortical lesions, but also due to the fact that the further discussed EI homeostasis mechanisms mostly play out in the cortex (Turrigiano, 2011). Therefore, while EI homeostasis may contribute to the recovery of connectivity between cortical regions after a stroke, its role regarding subcortical regions is not so clear.

In the face of the evidence toward widespread reorganization of cortical functional networks in the post-stroke cortex and its importance for the functional recovery of patients, underlying essential cognitive and motor adaptation, a fundamental question is then: how are these processes orchestrated globally through the cortex, given the localized nature of lesions? To answer that question, we propose that the fundamental tendency of neocortical networks to balance excitation and inhibition is a key participant in this effect. Since long-range projections in the human cortex are almost exclusively excitatory (Felleman and Van Essen, 1991; Tremblay et al., 2016), stroke lesions lead to an acute loss of excitation to distant areas, as encompassed by the original definition of diaschisis (von Monakow, 1914). Therefore, we hypothesize that this disruption will trigger the action of slow homeostatic plasticity mechanisms that adjust local synaptic weights to maintain balance, leading to changes in excitability and dynamics that in turn propagate throughout the brain. These widespread adaptations would then impact functional interactions between areas, participating in the recovery of the FC biomarkers mentioned in this section and in the appearance of new functional connections. Therefore, in the following sections, we summarize the findings regarding longitudinal and long-lasting changes in excitability in the post-stroke cortex. In addition, we highlight relevant findings showing EI balance as a fundamental property of cortical networks and the homeostatic plasticity mechanisms that actively work toward maintaining this equilibrium, emphasizing the correspondence between these processes and post-stroke variations in excitability.

## Diaschisis and Excitability

Due to the relative triviality of the concept of EI balance, it is not unreasonable to believe that anomalies to its normal function can be associated with pathologies such as stroke. To that effect, a parallel can be drawn between stroke and sensory deprivation studies (Hengen et al., 2013; Ma et al., 2019). Since most long-range connections in the cortex are excitatory (Felleman and Van Essen, 1991; Tremblay et al., 2016), the focal lesions experienced during a stroke effectively lead to a loss of excitation to regions that receive afferent terminals from the lesioned area, as encompassed by the original definition of diaschisis (von Monakow, 1914). Therefore, it is not far-fetched to think that these regions would experience EI balance changes similar to those described in sensory deprivation studies.

**FIGURE 2:**
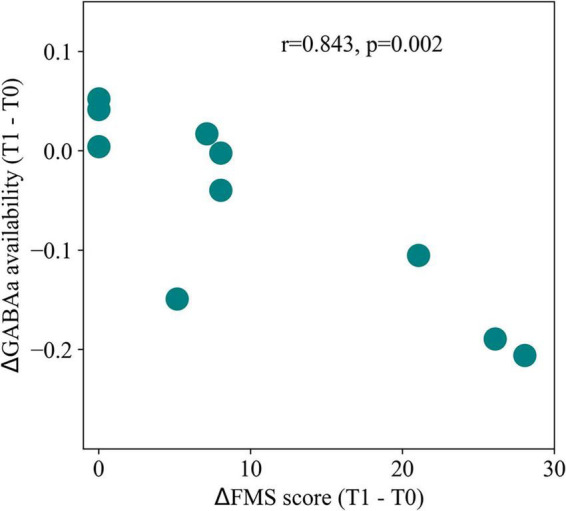
Post-stroke changes in inhibitory signaling. Correlation between motor recovery, quantified through Fugl-Meyer scores (FMS), and changes in GABAa receptor availability in the contralesional cortex, measured with positron emission tomography. Decreases in receptor availability were strongly correlated with improved motor function, illustrating the importance of increasing excitability through plasticity of inhibitory synapses for functional recovery. T0 and T1 represent 1 and 3 months post-stroke, respectively. Adapted from Kim et al. (2014).

In fact, there have been plenty of studies on changes in excitability experienced in the brain following a stroke (Murphy and Corbett, 2009; Carmichael, 2012). Neumann-Haefelin et al. (1995) first measured an increase in excitability in the peri-infarct cortex of rats, 7 days after induced ischemic lesion. In similar rodent studies, it was subsequently shown that hyper-excitability persisted in the cortex of ischemic rats for several months post-lesion (Luhmann et al., 1995), although other studies suggest it peaks in the first weeks post-lesion (Schiene et al., 1996). Furthermore, and while the initial study of Neumann-Haefelin et al. (1995) focused on perilesional areas (in the vicinity of an ischemic lesion), a subsequent study in rodents used a paired-pulse stimulation protocol to demonstrate that measurable changes extend to the contralesional cortex (Buchkremer-Ratzmann and Witte, 1997), indicating the presence of diaschisis. Later research in human stroke patients found a similar increase in cortical excitability of areas distant from the lesion, when compared to healthy controls (Butefisch, 2003). Importantly, such changes in excitability correlated positively with patient motor recovery, suggesting regulation of EI balance as a part of the process of functional recovery (Butefisch, 2003). Given the evidence supporting changes in the excitability of the post-stroke cortex, it is essential to understand the specific plastic process underlying such changes (Murphy and Corbett, 2009; Carmichael, 2012). A first candidate would be the regulation of excitatory signaling in post-synaptic pyramidal neurons. Indeed, research has shown it to be a possibility, manifested through prolonged excitatory postsynaptic potentials (EPSPs) mediated by N-methyl-D-aspartate (NMDA) receptors (Luhmann et al., 1995). When looking at receptor density instead, researchers found a gradual upregulation of NMDA receptors across the cortex of rats, lasting at least until 30 days post-lesion (Qü et al., 1999). In opposition to the idea of widespread increased excitability, a later study found no significant differences in the densities of both inhibitory γ-aminobutyric acid (GABA) and excitatory glutamate receptors in the cortex, apart from the perilesional area (Jolkkonen et al., 2003). Nonetheless, the same study showed a positive correlation between the contralesional number of binding sites for α-amino-3-hydroxy-5-methyl-4-isoxazolepropionic acid (AMPA), a glutamatergic receptor, and motor recovery after 25 days, suggesting a role of distant changes in excitability to the recovery process. Furthermore, Neumann-Haefelin and Witte (2000) showed a decreased amplitude of EPSPs in both ipsi and contralesional cortex, which would seemingly contradict the previously mentioned reports. However, the same researchers found a simultaneous downregulation of inhibitory synaptic transmission, which might compensate for the decrease in EPSP amplitude. In fact, changes in inhibitory signaling across the cortex seem to be even more involved in the post-stroke regulation of excitability than changes in excitatory transmission. Decreased inhibitory post-synaptic potentials (IPSPs) have been observed in rodent models of stroke (Luhmann et al., 1995) and found to also extend to regions in the contralesional cortex (Buchkremer-Ratzmann and Witte, 1997). Decreased inhibition was further measured in the affected hemisphere of stroke patients (Blicher et al., 2009), suggesting that this is not a detrimental effect, but part of the process of recovery, in accordance with subsequent results showing a positive correlation between decreased inhibition in the cortex of stroke patients and improved performance (Huynh et al., 2016). Such downregulation of inhibition has been mainly tied to changes in the density of GABAergic receptors. This decrease has been found across the brain already in the first-week post-stroke (Qü et al., 1998), remaining for several weeks post-lesion (Schiene et al., 1996; Redecker et al., 2002). Later, researchers have measured the availability of GABAa receptors in the cortex of stroke patients and found a similar longitudinal decrease 3 months after stroke (Kim et al., 2014). More importantly, the magnitude of this decrease correlated with the recovery of motor function (Figure 2). Conversely, changes in the number and structure of inhibitory neurons are not so clearly understood. While earlier studies found no structural changes in at least parvalbumin-positive interneurons (Luhmann et al., 1995), later results indicated a decrease in the number of both parvalbumin and somatostatin positive interneurons, visible at 30 days post-lesion (Alia et al., 2016). Nonetheless, it is clear that decreased inhibitory signaling across the brain is not only an essential

part of the observed changes in excitability following focal lesions, but also pivotal to the process of recovery. With all the evidence pointing toward the importance of the regulation of cortical excitability in stroke recovery, it is reasonable to assume that procedures aimed at manipulating the EI balance across the cortex can be applied to improve the behavioral outcomes of stroke patients. One of the possibilities is pharmacological manipulation. Some studies in this regard have focused on treatments that target, in particular, the increase in tonic inhibition in the acute period after stroke (Clarkson et al., 2010; Lake et al., 2015). While reducing tonic (or extrasynaptic) inhibition in the first days after stroke proved beneficial for recovery, it is also essential to focus on the potentiation of the processes previously mentioned in this section, which are more related to long-term changes in phasic inhibition. In that regard, it has been shown that reducing GABAa mediated transmission has prolonged effects, improving recovery up to 3 weeks after treatment (Alia et al., 2016). Pharmacological potentiation of AMPA receptors, in turn, has proved detrimental when applied at early stages, while a later application improved recovery, further supporting the delayed, long-term effect of these changes in excitability (Clarkson et al., 2011). Neuromodulation provides another option (Hummel and Cohen, 2006), and non-invasive techniques such as transcranial direct current (tDCS) and magnetic stimulation (TMS), aimed at increasing cortical excitability have been used to improve recovery in stroke patients (Blicher et al., 2009; Romero Lauro et al., 2014). An example of such techniques is theta burst stimulation (TBS) (Huang et al., 2005), based on the application of high frequency bursts of magnetic stimulation at intervals consistent with theta frequencies (i.e., ∼200 ms). The application of TBS has proved beneficial for the clinical recovery of stroke patients (Ackerley et al., 2010), particularly by enhancing motor learning when paired with other rehabilitation procedures (Meehan et al., 2011). Not only that, but TBS is thought to potentiate cortical excitability (Huang et al., 2005) and, when applied to stroke rehabilitation, can be used to stimulate increases in excitability of the ipsilesional cortex that relate to clinical recovery (Di Lazzaro et al., 2008; Ackerley et al., 2010; Boddington et al., 2020), although the specific contribution of each particular area (e.g., somatosensory and motor cortices) to motor recovery is not yet fully understood (Meehan et al., 2011). Interestingly, recent results suggest that TBS further acts on functional connectivity, contributing to the recovery of important properties such as interhemispheric connectivity and global efficiency (Ding et al., 2021). Taking that into consideration, it is reasonable to believe that mesoscale excitability and large-scale connectivity are synergetic processes and can be simultaneously modulated by the same techniques, fitting the hypothesis that EI homeostasis is an underlying driver of cortical reorganization.

On another note, concerns may be raised regarding possible incompatibilities between the results on cortical reorganization, mostly derived from resting-state imaging data from humans, and the bulk of research on post-stroke changes in excitability, which is performed in rodents. The latter is addressed in some of the studies in human data mentioned previously (Butefisch, 2003; Blicher et al., 2009; Kim et al., 2014; Huynh et al., 2016), where results in humans generally follow the ones obtained in rodent studies. In addition, recent results have shown that, with respect to FC, the changes observed after a stroke are comparable across species (Hall et al., 2021), further supporting the translation of findings from rodents to humans. Having established the current knowledge on the longitudinal changes in excitability that contribute to recovery from stroke, it is essential to understand the phenomena underlying these changes. In the next sections, we then discuss the importance of EI balance in cortical networks, given their circuit organization, and how it is maintained at a neuronal level by synaptic plasticity mechanisms.

## Excitatory-Inhibitory Balance in the Cortex

The cerebral cortex is the outer layer of neural tissue of the cerebrum of vertebrates, representing close to two-thirds of its volume. Cortical functions range from the integration of sensory information to higher cognitive functions such as attention, memory and consciousness (Kanwisher, 2010; Yeo and Eickhoff, 2016). The gross neuronal organization of cortical areas in the human brain is well known, comprising around 80% of excitatory neurons and 20% of inhibitory interneurons (Markram et al., 2004; Tremblay et al., 2016). Excitatory neurons are thought to be primarily responsible for coding and communicating messages across the cortex, since long-range cortical connections are almost exclusively excitatory (Felleman and Van Essen, 1991; Tremblay et al., 2016). In contrast, inhibitory interneurons form local and densely interconnected networks (Fino and Yuste, 2011; Tremblay et al., 2016). Altogether, these connectivity patterns lead to single pyramidal neurons in the cortex receiving thousands of excitatory and inhibitory inputs from other neurons. In fact, early studies attempting to define the canonical cortical microcircuit in a layer-specific manner (Douglas et al., 1989) already hinted at the fact that excitatory and inhibitory activity are not independent phenomena, but arise in a synergetic manner. At a relatively simplified level, the interplay between excitatory and inhibitory inputs unto pyramidal neurons and, in particular, their specific temporal patterns, causes postsynaptic neurons to fire action potentials when excitation surpasses inhibition sufficiently to reach the firing threshold. To add a level of complexity to the microcircuit organization of cortical networks, cortical areas connect to each other through long-rage excitatory white matter projections, the organization of which is far from random. Research shows that cortical networks organize in a small-world manner (Achard et al., 2006; Bassett and Bullmore, 2006), being highly clustered, while maintaining relatively short paths between their elements. In this architecture, a small number of highly interconnected areas serve as hubs of communication across the cortex, for which reason cortical connectivity is sometimes referred to as a rich-club (van den Heuvel and Sporns, 2011). In addition, tracing data from the macaque cortex (Markov et al., 2013), which gives information not only about strength, but also directionality of connectivity, suggests that the cortex organizes in a bow-tie manner, with a densely connected core sitting in the middle of the stream of information in the cortical hierarchy. In any case, it is relevant to consider that cortical areas receive varied levels of incoming excitation due to these differences in connectivity. While the initial canonical view of Douglas et al. (1989) states that most of the excitation received by pyramidal neurons is from local recurrent connections, the stipulated architectural differences in connectivity, and thus in incoming excitation, are still meaningful. Because the function of not only individual neurons, but also local neuronal networks in the cortex, is susceptible to imbalances in the excitatory and inhibitory inputs, it is then relevant to study how these inputs are balanced across the cortex. In their seminal 1996 work, van Vreeswijk and Sompolinsky (1996) demonstrated that a state of EI balance emerged spontaneously in artificial networks of coupled excitatory and inhibitory neurons, when connections were sparse and strong. Importantly, in such networks, the activity of individual neurons was dependent on random fluctuations in these inputs and consistent with the irregular activity empirically observed in cortical neurons. In the past 20 years, further modeling studies have stressed the importance of EI balance to realize specific computational principles of cortical networks. In particular, balanced networks of excitatory and inhibitory neurons ensure stochastic fluctuations in synaptic drive, which reproduce the irregular firing patterns observed *in vivo* and impact how neurons react to external noise, rendering them more sensitive to correlated input (Salinas and Sejnowski, 2000). In addition, it has been shown that appropriate EI balance at a neuronal level, in recurrent networks, simultaneously leads to increased robustness to input and output noise (Rubin et al., 2017). Furthermore, it has also been postulated that a possible solution for the correct transmission of rate-coded information through layers in feedforward networks would be to have balanced feedback inhibition (Litvak et al., 2003), in accordance with the principles initially explored by van Vreeswijk and Sompolinsky (1996). Nonetheless, studies regarding the role of EI balance in cortical function have not been limited to the computational realm. Research in the auditory (Wehr and Zador, 2003; Froemke et al., 2007), barrel (Okun and Lampl, 2008), and visual (Mariño et al., 2005; Xue et al., 2014) cortices shows that the inhibitory input to pyramidal neurons tightly matches excitation and suggests that this property contributes not only to avoid runaway activity, but also to a sharper tuning of neurons to external stimuli. Critically, this state of balanced excitation and inhibition arises during neural development, in parallel to characteristics such as the aforementioned sensory co-tuning, in both the auditory (Dorrn et al., 2010) and visual cortices (Tao and Poo, 2005). In addition, the maintenance of EI balance is likely not limited to the sensory realm, being crucial for higher brain functions such as memory, by helping maintain stable activity and avoiding interference during learning of new memory traces (Vogels et al., 2011; Litwin-Kumar and Doiron, 2014). Furthermore, the maintenance of EI balance has been observed to occur across cortical states, such as sleep-wake cycles (Hengen et al., 2013) and quiescence vs. active locomotion (Zhou et al., 2014), although recent studies seem to indicate a more complex picture of differential points of balance being maintained in sleep and wake states (Bridi et al., 2020). Interestingly, in electrophysiological data from both human and monkey cortex, Dehghani et al. (2016) found a co-fluctuation of excitatory and inhibitory signals which is not only maintained through different stages of the sleep-wake cycle, but also present when looking at the detain a wide range of timescales. To further add to the importance of EI balance in cortical networks, a number of disorders such as epilepsy (Kullmann et al., 2012; Dehghani et al., 2016) schizophrenia (Kehrer et al., 2008; Kullmann et al., 2012), depression (Concerto et al., 2013; Page and Coutellier, 2019), anxiety (Page and Coutellier, 2019), chronic stress (Page and Coutellier, 2019), Alzheimer’s disease (Vico Varela et al., 2019; Kazim et al., 2021), and autism spectrum disorders (Nelson and Valakh, 2015; Bruining et al., 2020), have been associated with alterations in cortical EI balance or excitability, supporting the idea that proper maintenance of this regime is crucial to ensure the correct function of the cortex.

All that considered, one should address the fact that the idea of a tight balance between excitation and inhibition seems to go against previous knowledge on the structure and function of cortical networks. Firstly, there is evidence that inhibitory interneurons form dense and largely unspecific networks on a local scale (Fino and Yuste, 2011; Packer and Yuste, 2011), which, at least, rules out the hypothesis that matching excitation and inhibition in individual neurons emerges from precise structural properties of connectivity. In addition, even though the blueprint of cortical microcircuitry is relatively stereotypical across the brain (Markram et al., 2004; Tremblay et al., 2016), local heterogeneities in the number of neurons and the relative proportion of excitatory and inhibitory synapses (Wang, 2020) add an extra layer of complexity to a tight maintenance of balanced activity. However, either by showing that excitatory and inhibitory postsynaptic currents (Xue et al., 2014) or conductances (Mariño et al., 2005) are matched in magnitude in individual neurons, or that there exists a precise stimulus co-tuning of excitatory and inhibitory synapses in individual pyramidal neurons (Wehr and Zador, 2003; Froemke et al., 2007), the corpus of research mentioned previously seems to point toward a tightly maintained EI balance, at the very least in sensory areas. Furthermore, the early study of Vreeswijk and Sompolinsky assumed the existence of a random, homogeneous, and sparse connectivity, leading to a natural emergence of EI balance (van Vreeswijk and Sompolinsky, 1996). However, due to phenomena such as the formation of neuronal assemblies through Hebbian plasticity (Denève and Machens, 2016; Mackwood et al., 2021), cortical networks are seldomly homogeneous, challenging the applicability of Vreeswijk and Sompolinsky’s principles. Not only that, but the locally dense nature of inhibitory connectivity in the cortex (Fino and Yuste, 2011; Packer and Yuste, 2011) renders the assumption that balanced networks need to be sparse for EI balance to emerge unrealistic. Conveniently, these drawbacks have been addressed recently. Firstly, it was shown that the conclusions would still hold in networks with dense connectivity, as long as synaptic weights scale with the numbers of connections (Barral and Reyes, 2016). Then, in recurrent networks with introduced inhomogeneities in incoming connectivity, it was demonstrated physiologically relevant patterns of activity could be achieved as long as excitatory and inhibitory in-degrees were matched in individual neurons (Landau et al., 2016).

Considering all the evidence, the importance of having balanced excitatory and inhibitory inputs at a neuronal level seems clear. However, the influence of this balance spreads beyond the cellular scale into the realm of network activity patterns. In that regard, another aspect of the human neural function where EI balance is considered to play a fundamental part is the concept of criticality. Criticality, as a theory, was borrowed from physics and it pertains to an operating point in systems of interacting units on the verge of transitioning between states (Beggs and Timme, 2012). Importantly, criticality stipulates a dependence of the transition between phases on a control parameter, which is often related to EI balance. By analyzing trademarks of criticality such as the size and duration distribution of neural avalanches in resting-state activity, researchers have been able to detect it ubiquitously in the mammalian brain. From the first study of spike data in the rat somatosensory cortex *in vitro* by Beggs and Plenz (2003), signatures of criticality have been detected in cortical slices (Friedman et al., 2012) and cultures (Pasquale et al., 2008), *in vivo* data from the cortex of rats (Ma et al., 2019), cats (Hahn et al., 2017), and monkeys (Petermann et al., 2009; Hahn et al., 2017) and oscillatory data from human subjects (Poil et al., 2008; Bruining et al., 2020). This constant presence of criticality in brain systems suggests some degree of functional advantage. Indeed, the maintenance of activity near the critical transition point has been speculated to optimize crucial functional characteristics of neural networks such as dynamic range (Shew et al., 2009), information capacity and transmission (Shew et al., 2011) and computational power (Beggs, 2008).

## Plasticity Mechanisms for Excitatory-Inhibitory Homeostasis

**FIGURE 3:**
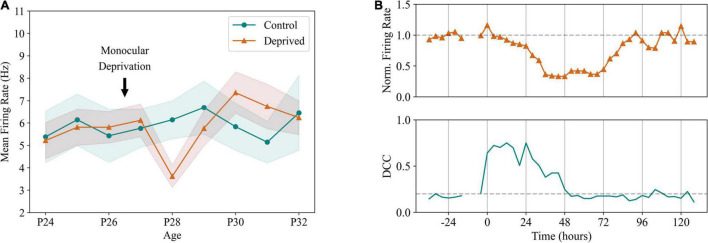
Firing rate homeostasis in the cortex. **(A)** Homeostatic regulation of pyramidal neuron firing rates in a sensory deprivation protocol. In this experiment, unilateral deprivation of visual input was performed in rodents through lid suture, late at P26. Firing rates of the visual cortex in the deprived hemisphere show a significant reduction in the second day following suture, being recovered to pre-deprivation levels after 72 h, an effect not visible in the unaffected hemisphere. Adapted from Hengen et al. (2013). **(B)** After monocular deprivation, dynamics of the affected hemisphere show an immediate departure from criticality, quantified by increased distance from criticality coefficients (DCC). Criticality was recovered after 48 h through homeostatic plasticity, preceding the recovery of excitatory firing rates and indicating criticality as a setpoint for cortical dynamics. Adapted from Ma et al. (2019).

Considering the evidence that EI balance is a pivotal characteristic of normal cortical activity, it is natural to question how it is maintained in the face of varying external inputs, changing behavioral states and different levels of incoming excitation due to the structural patterns of cortical connectivity (Bassett and Bullmore, 2006; van den Heuvel and Sporns, 2011; Markov et al., 2013). The logical assumption would then be that cortical neurons are equipped with plasticity mechanisms capable of maintaining a balance between excitatory and inhibitory incoming activity (Sprekeler, 2017). Indeed, homeostasis of EI balance has been a topic of study in the past years. Turrigiano et al. (1998) demonstrated that pyramidal neurons scale the amplitudes of excitatory postsynaptic currents when grown in cultures where either excitatory or inhibitory signaling was altered. This type of plasticity, likely occurring postsynaptically, was dubbed synaptic scaling. Operating at long timescales (hours to days in rodents) (Turrigiano, 2011), synaptic scaling facilitates the homeostatic maintenance of stable firing rates in pyramidal neurons without disrupting the fluctuations occurring in faster timescales essential for encoding and communicating information across the brain. In general, sensory deprivation studies have been the most common procedure to assess firing rate homeostasis *in vivo*. Commonly performed in rodents, sensory deprivation aims at disrupting the normal levels of excitation arriving at the primary visual cortex by interrupting connectivity from the retina. This is achieved mainly by suturing one of the eyelids (Maffei et al., 2004; Maffei and Turrigiano, 2008; Hengen et al., 2013; Ma et al., 2019), although retinal lesion (Keck et al., 2013) or inhibition of optic nerve firing (Maffei et al., 2004) have been applied to the same purpose. In all of the mentioned studies, neuronal firing rates in sensory cortices showed a decrease in activity, which was subsequently restored to baseline levels within a timescale of days (Figure 3A). A recent study further elucidated on these processes by showing that cortical networks *in vivo* depart from criticality after sensory deprivation and likely return to it through synaptic scaling, suggesting criticality as a possible homeostatic target in cortical networks, in parallel with stable firing rates (Figure 3B; Ma et al., 2019). The relevance of this work lies mainly in the fact that it provides a well-defined activity target regime for firing rate homeostasis, which goes toward the problem of conceptualizing and defining an optimal baseline firing rate that should be maintained to ensure proper cortical function.

While Turrigiano et al. (1998) and Turrigiano (2011) first hypothesized that synaptic scaling manifests mainly through postsynaptic changes in excitatory synapses, further research suggests that EI homeostasis may involve the regulation of inhibitory synapses unto pyramidal neurons or even their intrinsic excitability (Desai et al., 1999; Maffei and Turrigiano, 2008). However, the latter is not always observed (Maffei et al., 2004) and is thought to only come into play when scaling of synapses fails to compensate for perturbations (Turrigiano, 2011). As for inhibitory plasticity, the main arguments in favor of it being one of the main drivers of EI balance homeostasis lie in the fact that, in pyramidal neurons of developing sensory systems, the tuning curves of inhibitory postsynaptic currents evolve to match their excitatory counterparts closely (Dorrn et al., 2010) and change accordingly when the tuning of excitatory connections is altered (Wehr and Zador, 2003; Froemke et al., 2007). In addition, reducing excitability in layer II/III pyramidal neurons did not lead to changes in excitatory synapses, but to decreased inhibition from fast-spiking interneurons instead (Xue et al., 2014). More importantly, this effect was dependent on the activity of the postsynaptic target, fitting the characteristics of synaptic scaling. Studies in hippocampal pyramidal neurons seem to indicate an additional dependence of synaptic scaling of inhibitory synapses on presynaptic activity, as opposed to their excitatory counterparts, which only depend on post-synaptic activity (Turrigiano, 2011). Therefore, the pre and post-synaptic dependence of inhibitory synaptic scaling renders it more sensitive to changes in network activity, as opposed to excitatory synapses, which are thought to be scaled only accordingly to post-synaptic activity, in a cell-autonomous process. This, again, stresses the importance of inhibitory plasticity in the maintenance of EI balance at a network level. Accordingly, Vogels et al. (2011) derived a homeostatic inhibitory plasticity rule dependent on both pre and postsynaptic activity, aimed at maintaining postsynaptic firing at a defined target rate. In their study, this plasticity mechanism was sufficient to replicate a range of phenomena such as co-tuning of excitatory and inhibitory synapses, sparse responses and a return of balance and co-tuning after perturbation. In recurrent neural networks, a similar rule rendered dynamics robust to small perturbations, while still allowing for the formation and rearrangement of neural assemblies with sufficiently strong inputs (Litwin-Kumar and Doiron, 2014). Recently, a comparable form of synaptic plasticity was analytically derived in a recurrent neural network model (Mackwood et al., 2021), which simultaneously posed the hypothesis of similar processes happening in excitatory synapses unto inhibitory neurons. In fact, while most of the attention has been devoted to pyramidal neurons, studies have shown that changes in fast-spiking interneurons may also contribute to the maintenance of EI balance, either through regulation of intrinsic excitability or of incoming excitatory synapses (Kullmann et al., 2012).

All considered, it can be said that EI balance seems to be crucial to the function of cortical networks, that there are mechanisms in place to actively maintain this balance and that anomalies in the equilibrium are associated with a series of pathologies affecting a broad range of functions. That said, future studies should further focus on the hypothesis of criticality as a homeostatic target for cortical dynamics and how individual neurons capture departures from this regime and translate them into changes in synaptic dynamics or intrinsic excitability. In addition, attention should be devoted to the possibility of synaptic scaling mechanisms in cortical inhibitory interneurons playing an important role in regulating cortical dynamics, synergizing with similar processes in their excitatory counterparts.

## Tying Excitatory-Inhibitory Homeostasis and Functional Reorganization in Stroke Recovery

The longer timescale of post-stroke changes in excitability and the occurrence of processes such as the up or downregulation of glutamatergic and GABAergic receptors (Luhmann et al., 1995; Qü et al., 1998, 1999; Neumann-Haefelin and Witte, 2000; Kim et al., 2014) suggests a connection between stroke and the homeostatic mechanisms of synaptic scaling (Turrigiano, 2011). Indeed, this possibility has been suggested before (Murphy and Corbett, 2009; Platz, 2021) and the current knowledge on EI homeostasis seems to fit the hypothesis that the general increase in excitability experienced in the post-stroke cortex might be a homeostatic response to a sudden loss of excitation from lesioned areas. A further possible connection to cortical reorganization can be made, given that FC has been previously tied to local excitability in both stroke patients (Volz et al., 2015) and healthy subjects (Nettekoven et al., 2015), offering a possible framework to tie the meso (local EI balance) and macro (global changes in network dynamics) scales of diaschisis. That said, we hypothesize that EI homeostasis, manifesting at a local level in cortical networks through mechanisms like synaptic scaling, is one of the main drivers of the process of recovery from diaschisis in the cortex of stroke patients. More importantly, the role of EI homeostasis is likely not limited to the adaptation of cortical regions to the loss of excitability experienced after a lesion in distant areas, but it may also be an important player in the process of cortical reorganization and recovery of FC properties relevant for recovery (Wang et al., 2010; Siegel et al., 2016, 2018; Griffis et al., 2019). This approach brings forward many interesting possibilities for exploration. First, an important concern that is rarely addressed by studies in excitability and stroke recovery is the putative side effects of compensatory changes in local EI balance. It is evident that proper EI balance is critical for the correct function of cortical networks (Beggs, 2008; Vogels et al., 2011; Xue et al., 2014; Rubin et al., 2017) and imbalances have been associated with several neural pathologies, some of which associated with stroke. A particular case is epilepsy (Kullmann et al., 2012; Dehghani et al., 2016) and, in a study with a rodent stroke model, it was demonstrated that, even though homeostatic reduction of tonic inhibition in the motor cortex proved to be beneficial to the recovery of motor function, it also raised the risk of suffering from epileptic seizures (Jaenisch et al., 2016). Such findings raise a concern about possible side effects coming from the recovery of cortical function, which is of particular importance, in this case, given that epilepsy is a common side-effect of stroke (Feyissa et al., 2019). While no similar studies have been performed in relation to the slow changes in phasic inhibition, it should not be out of the question that they might increase the propensity for the development of late-onset side effects related to EI balance, such as depression (Towfighi et al., 2017) in a patient-specific manner. That said, emphasis should be placed on relating recovery of FC and function to the appearance of side-effects of stroke that can be associated with EI imbalances, such as depression (Concerto et al., 2013; Towfighi et al., 2017; Page and Coutellier, 2019), fatigue (Kuppuswamy et al., 2015), chronic pain (Klit et al., 2011; Ratté and Prescott, 2016), and epilepsy (Feyissa et al., 2019).

Another particular direction of research is to investigate how targeted stimulation of particular areas in the brain might be used to tackle specific post-stroke deficits and how this effect ties to the regulation of local excitability through neuromodulation. While approaches such as tDCS have been traditionally applied to modulate excitability, our hypothesis suggests that such neuromodulatory therapies may also be applied to stimulate cortical reorganization and the recovery of FC properties to healthy levels. Indeed, connectivity can also be modulated with neurostimulation techniques usually targeted at regulation of excitability (Grefkes and Fink, 2012). TBS has been shown to modulate both excitability (Ackerley et al., 2010; Boddington et al., 2020) and connectivity (Ding et al., 2021) in stroke patients and healthy controls (Nettekoven et al., 2014), although results in the latter showed that, even though both processes happen simultaneously, a correlation between them could not be found.

While it is well established that neuromodulation can be used to improve functional recovery in stroke patients (Blicher et al., 2009; Romero Lauro et al., 2014), we suggest that a deeper understanding of its effects not only in excitability, but also FC, is essential to unlocking the full potential of these rehabilitation procedures. In addition, the use of adaptive systems, such as the Rehabilitation Gaming System (RGS) (Cameirão et al., 2010), has been shown to engage widespread areas across the human cortex (Prochnow et al., 2013) and stimulate cortical reorganization of motor networks (Ballester et al., 2017), being a further potentiator of FC recovery. That said, we suggest an additional focus for future research on the synergy between the use of such adaptive rehabilitation procedures and neuromodulation techniques, in order to better potentiate beneficial changes in both excitability and connectivity.

Finally, it should not be out of the question that stroke is accompanied by impairments in EI homeostasis in the cortex. Data shows that the extent of changes in excitability correlates with recovery (Murphy and Corbett, 2009; Kim et al., 2014), suggesting that such impairments are likely to happen and impact the adaptive ability of the post-stroke brain. In this context, understanding the link between global changes in FC and local excitability would be a valuable contribution, by allowing the inference of localized disrupted homeostasis from global connectivity metrics extracted from non-invasive techniques such as fMRI. With this information, neuromodulation therapies could then be applied in a more targeted manner to areas with particularly impaired EI balance in a patient-specific manner. Again, we stress that the benefits of such individualized therapies would not be limited to excitability, likely extend toward a better recovery of functional connectivity.

**FIGURE 4:**
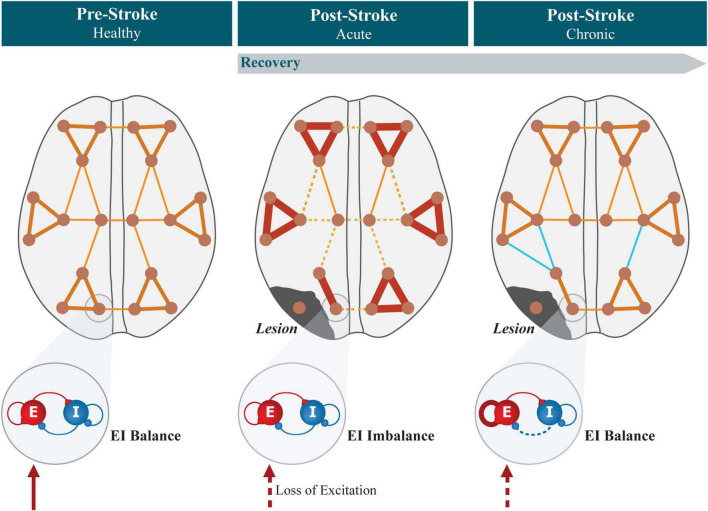
Summary of the evolution of connectivity and local EI balance in cortical networks after stroke. In the healthy brain, functional networks show a modular structure, with strongly connected regions forming modules, connected by functional hubs. Excitatory and inhibitory synapses are regulated locally by EI homeostasis so that balance is maintained, accounting for the level of excitation received from external sources and keeping neuronal firing rates stable. Focal lesions experienced by stroke patients lead to neural tissue necrosis in confined areas. In the acute period after lesion, the modular organization of functional networks is disrupted, showing increased segregation between modules, together with decreased interhemispheric and increased intrahemispheric connectivity. Simultaneously, areas that previously received white matter projections from the lesioned region experience a sudden reduction in the levels of external excitation, causing a local imbalance in excitatory and inhibitory activity. During the process of recovery, synaptic plasticity compensates for the reduced excitation by increasing/decreasing the strength of excitatory/inhibitory synapses unto pyramidal neurons, increasing their excitability to restore local EI balance. Concurrently, functional connectivity changes toward pre-lesion levels, either through the recovery of healthy functional networks or *via* cortical reorganization and the formation of new functional pathways (represented in light blue).

That considered, it is first and foremost essential to address the relationship between post-stroke changes in excitability and cortical reorganization (summarized in Figure 4), scanning for putative causal relations between both phenomena, possible impairments in EI homeostatic mechanisms such as synaptic scaling (Turrigiano, 2011) and their further suitability as a target for post-stroke rehabilitation procedures addressing excitability and connectivity concurrently. However, studying both aspects of cortical function simultaneously and with the needed level of detail might be a difficult endeavor when analyzing human imaging or electrophysiological data. This is due to the different scales of both processes, which would require the use of different imaging or recording modalities or advanced techniques of inferring excitability from techniques such as fMRI. This is where large-scale computational modeling becomes a useful framework for studying the evolution of whole-brain dynamics after disruptive events. In the next section, the state of the art of large-scale modeling will be described, particularly in the topics of EI homeostasis and the evolution of cortical networks in reaction to stroke/localized lesions.

## Large Scale Modeling of the Human Cortex: Excitatory-Inhibitory Balance and Stroke

Computational modeling is a powerful tool for the exploration of neural dynamics, allowing for a more direct analysis and easier parameter manipulation than physiological studies. One can achieve this by applying mathematic concepts to the description of neural dynamics at different scales, from the molecular and synaptic level to the modeling of large-scale activity. The latter relies on the assumption that the activities of individual neurons are generally uncorrelated, meaning that the ensemble activity can be reduced to its mean and variance (Breakspear, 2017). This mean-field approach can be extremely useful in computational neuroscience, allowing for the modeling of large-scale dynamics at a higher level of abstraction, which is more computationally tractable. Since the model of interacting excitatory and inhibitory populations derived by Wilson and Cowan (1972), different mean-field models have been derived and applied in the study of large-scale dynamics of the cortex (see Breakspear, 2017 for a detailed review). While neural mass models can accurately describe, with some level of abstraction, the behavior of local cortical populations of interacting excitatory and inhibitory populations at a mesoscale, large-scale cortical dynamics require a further level of complexity. To bridge the gap between scales, one can look at the cortex as a network of connected local neural masses. In that regard, great progress is due to the advent of Connectomics, coined by Sporns et al. (2005) to describe the anatomical connections between regions in the human brain. While large scale connectivity has been a topic of exploration in primates already since the nineties (Felleman and Van Essen, 1991), human Connectomics have been a particular field of interest in the past 15 years, after the consolidation of the standard approach to human brain mapping by unraveling the structural core of the human cortex using diffusion spectrum imaging (Hagmann et al., 2007, 2008). This step allowed for the investigation of the graph properties of the human cortex, finding relations between structural connectivity (SC) and FC (Sporns, 2011; Thomas Yeo et al., 2011; Goñi et al., 2014). Honey et al. (2007) showed that simple mean-field models constrained by macaque SC data could reproduce features of empirically measured resting-state FC. Later, through a similar approach, the same researchers demonstrated, with a higher resolution human connectome, how SC shaped large-scale dynamics and can inform properties of FC (Honey et al., 2009). Since then, several whole-brain modeling studies have contributed to our knowledge about how model parameters influence large-scale dynamics (Deco et al., 2009), how clusters of regions in the human cortex synchronize and interact to form large-scale networks (Cabral et al., 2011) and how these interactions underlie certain cognitive functions and processes (Thomas Yeo et al., 2011).

Moreover, EI balance has also been in the scope of research in whole-brain modeling. The reliance of approaches such as the Wilson-Cowan model on interacting excitatory and inhibitory populations, with a relatively simple parameter space, provides a highly controllable and measurable framework to study the effects of EI balance manipulations. Deco et al. (2014) conducted one of the first large-scale modeling studies to investigate how changes in local EI dynamics impact global properties of cortical networks. By applying a regulatory mechanism on local feedback inhibition aimed at maintaining excitatory firing rates stable (similar to synaptic scaling; Turrigiano, 2011; Vogels et al., 2011) they demonstrated that models with self-maintained local EI balance better predicted empirical FC for a wider range of parameters and showed improved information capacity. Later, Hellyer et al. (2016) adapted the inhibitory mechanism proposed by Vogels et al. (2011) into a large-scale model of the human cortex. In this model, the parameters of local populations were such that, depending on the input levels, the dynamics experienced a Hopf bifurcation, transitioning from a state of irregular, noise-driven low activity, to an oscillatory one. When using this transition point as a homeostatic target for inhibitory plasticity, models achieved a better fit to empirical FC and maintained criticality across the cortex. Furthermore, it was shown that local inhibition strength was correlated with graph properties of the connectome, further emphasizing how local and global properties interact to shape cortical activity. More importantly, by exploring the homeostatic firing rate target, the authors emphasize the importance of poising local activity near the critical Hopf bifurcation, stressing the relationship between EI balance, criticality and cortical function (Ma et al., 2019). Building on these foundations, further research demonstrated that the same homeostatic mechanisms rendered cortical networks more robust to changes in parameters such as transduction delays and global coupling strength (Abeysuriya et al., 2018). Interestingly, in a recent study, using an updated model of coupled excitatory and inhibitory populations with a term that quantifies the concentration of excitatory and inhibitory neurotransmitters at a local level, it was found that there are optimal concentrations of glutamate and GABA that maximize fit of simulated and empirical FC (Naskar et al., 2021). Not only that, but these suggested optimal concentrations also corresponded to a working point of activity where the brain is in a state of heightened metastability. Importantly, by changing the concentration of neurotransmitters to simulate epilepsy conditions, they could reproduce findings regarding the segregation and integration of FC graphs. In this case, epilepsy was used as a use case, but similar measures have been applied in stroke (Honey et al., 2007; Philips et al., 2017; Zhang et al., 2017; Siegel et al., 2018), which points toward the utility of applying such models to study brain pathologies in general. Furthermore, the regional heterogeneities in cortical microcircuitry (Wang, 2020) have also been brought to the field of computational modeling (Deco et al., 2021). By varying the excitability of networks nodes according to physiological constraints, it was shown that the system was able to reproduce phenomena like ignition dynamics and a hierarchy of intrinsic timescales (Wang, 2020), while maintaining the similarity between simulated and empirical resting-state FC. While this perspective might first seem at odds with the previous works of Hellyer et al. (2016) and Abeysuriya et al. (2018), which consider local cortical regions to regulate toward the same setpoint of criticality across the brain, the model in question also included a mechanism akin to synaptic scaling to maintain the firing rate of populations clamped at a physiologically realistic level, as in previous work (Deco et al., 2014). This model hints at the possibility of reconciling the maintenance of cortical activity at criticality (Ma et al., 2019), while simultaneously allowing for functional specialization (Mashour et al., 2020). Nonetheless, it did not capture particular aspects of ignition dynamics, such as higher-order areas (e.g., prefrontal cortex) maintaining self-sustained activity after stimulus termination (Mashour et al., 2020), which remain to be investigated. Therefore, further studies should elaborate on how homeostatic plasticity acts in the face of structural heterogeneities, the possibility of different local targets for EI balance and possible regulation through neurotransmitters. In addition, importance should be given to reconciling the hypothesis of criticality as an optimal working point across the cortex and the functional specialization of specific cortical areas, particularly higher-order ones.

Whole-brain modeling has also proved useful in a more applied context, in neurological disorders such as epilepsy (Jirsa et al., 2017), Alzheimer’s (Stefanovski et al., 2019) and Parkinson’s disease (Saenger et al., 2017), and respective exploration of therapeutical procedures (Deco and Kringelbach, 2014). This has also been the case for the effects of local lesions and stroke, in particular. While earlier studies had already assessed the effect of node removal when analyzing brain functional networks (Achard et al., 2006) and structural (Sporns et al., 2007; Gratton et al., 2012) networks, Honey and Sporns first modeled the effect of localized lesions in a whole-brain model of the macaque cortex (Honey and Sporns, 2008). In their study, lesions to structural hubs of the connectome lead to larger changes in system dynamics, spread beyond the vicinity of lesions, akin to the phenomenon of diaschisis (Carrera and Tononi, 2014). Subsequent studies in the human connectome (Alstott et al., 2009) further elaborated on which networks are more vulnerable to damage and how the graph properties of SC can help predict the effects of targeted lesions. In addition, further research has delved into how localized lesions affect network graph properties (Cabral et al., 2012; Joyce et al., 2013). Nonetheless, research in this field has not been confined to studies of the effect of lesions in networks obtained from healthy controls. In a subsequent modeling study (Falcon et al., 2016), large-scale models were constrained with DTI data from stroke patients and healthy volunteers to evaluate the fit to empirical FC by changing parameters such as global coupling, conduction delays and local EI coupling. The authors found that stroke patients had, among other features, reduced inhibitory to excitatory coupling compared to healthy controls, similarly to previous results (Kim et al., 2014) and in line with the hypothesis of synaptic scaling of inhibitory synapses (Turrigiano, 2011; Vogels et al., 2011; Hellyer et al., 2016; Abeysuriya et al., 2018). While the decreased inhibition did not correlate with motor performance, the coupling from excitatory to inhibitory neurons showed a negative correlation, which is still in line with the hypothesis of increased excitability. This latter effect is due to decreased excitation to interneurons, translating into weaker feedback inhibition. While evidence toward synaptic scaling of E to I synapses is not so clear (Kullmann et al., 2012), it might also occur as a mechanism of EI homeostasis (Turrigiano, 2011), having, therefore, a possible role in stroke recovery deserving further investigation. On another note, Adhikari et al. (2017) used an algorithm to adjust modeled structural connectivity to optimize the fitting between empirical and functional networks and found that changes in information capacity and integration, graph properties relevant in the context of stroke (Philips et al., 2017; Zhang et al., 2017; Siegel et al., 2018), are not a direct consequence of lesion volume. When looking at changes at a resting-state network level (Thomas Yeo et al., 2011), a generalized decrease in the level of integration and information capacity has been reported, with the latter being significantly correlated with impairments in network-specific functions (Adhikari et al., 2017).

**FIGURE 5:**
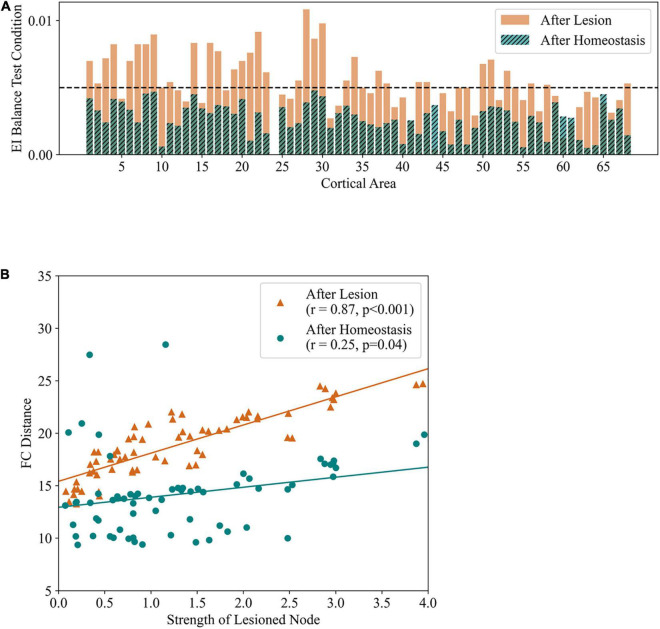
EI homeostasis and recovery of FC cooccur in large-scale computational models of stroke. Results from the application of focal structural lesions in a large-scale model of the cortex with local EI homeostasis. **(A)** EI balance test condition measured immediately after lesion in the left precuneus and after homeostatic adaptation. Areas with values lower than 0.005 are considered balanced. It can be observed that EI balance is lost across the cortex immediately after lesion, being re-established by local homeostasis. **(B)** Correlation between the strength of lesioned nodes and differences in FC from baseline (FCD), immediately after lesion (black) and after restoring EI balance (black). Adapted from Vattikonda et al. (2016).

A common shortcoming of the modeling studies on the effects of lesions mentioned above is that, while network activity is dynamic, the model parameters are either static or manipulated to improve fitting with empirical data. In contrast, cortical networks are plastic and undergo observable changes after focal lesions, particularly in the case of stroke, either in terms of connectivity (Wang et al., 2010, 2014; Siegel et al., 2018) or excitability (Kim et al., 2014; Huynh et al., 2016). The existence of plasticity mechanisms for EI homeostasis and the parallel between stroke and sensory deprivation (Hengen et al., 2013; Ma et al., 2019) bring into light the importance of studying how plastic whole-brain networks react in the face of localized lesions. Studies tackling this particular challenge have been scarce and show limitations in their approach. Vattikonda et al. (2016) were the first to study how EI balance homeostasis may aid in post-stroke recovery, in a model where homeostatic plasticity of local inhibitory synapses was applied through a recursive process aimed at maintaining firing rates at a physiological level (Figure 5A). They found that, even though the immediate effects of lesions were strongly dependent on the strength and participation coefficient of lesioned nodes, EI homeostasis was able to return resting-state FC close to pre-lesion levels. Furthermore, the success of recovery was dependent on lesioned node strength, indicating that the level of recovery attained might depend on the properties of the lesioned area, albeit less strongly than the immediate effects of lesions (Figure 5B). In particular, these results showed a simultaneous recovery of functional connectivity and EI balance, although no particular correlation was established between the two. Despite the fact that the mathematical formulation of plasticity was, in this case, different from more physiologically grounded rules (Vogels et al., 2011), the working principle is quite similar to synaptic scaling and, particularly, to other implementations in whole-brain modeling (Hellyer et al., 2016; Abeysuriya et al., 2018). Later, a similar study was performed in conjunction with behavioral analysis of longitudinal data from stroke patients (Rocha et al., 2020). Homeostatic plasticity was implemented through a normalization of the weights of the structural connectivity matrix after a number of simulation timesteps, thus keeping the levels of incoming excitation to each cortical region relatively constant across time. The authors found that dynamic properties of cortical networks, such as criticality, are altered after focal lesions and slowly recovered in parallel with behavior, pointing to a group of structural connections important for the recovery of criticality, mainly related to the default mode, attention and execution networks. While having the advantage of being a patient-specific study, its shortcomings lie in the used plasticity mechanism. Firstly, it was dependent on post-synaptic weights, and not on activity, while synaptic scaling of excitatory synapses shows dependence on postsynaptic activity instead (Ibata et al., 2008; Turrigiano, 2011). Secondly, plasticity was applied on the long-range structural excitatory connections, not accounting for the important role of inhibitory plasticity in EI homeostasis (Turrigiano, 2011; Vogels et al., 2011; Xue et al., 2014) and the fact that the excitatory connectivity of excitatory neurons is mostly received from local sources (Douglas et al., 1989; Hellwig, 2000). In other words, the most significant effect of excitatory synaptic scaling would be felt in local recurrent connections. Nonetheless, the study was not targeted in particular to the study of the role of local synaptic scaling in EI homeostasis, providing, still, useful insights into altered post-stroke dynamics and the evolution of large-scale structural connectivity.

Finally, a common shortcoming of all the mentioned models is the lack of a deeper investigation of the side-effects of the recovery of functional connectivity by homeostatic mechanisms, particularly regarding changes in the excitability of particular regions, which might be tied to known side effects of stroke, as already hinted about by previous research (Jaenisch et al., 2016). That said, further attention should be devoted to modeling how EI balance evolves after perturbations such as stroke lesions. Studies should consider the inclusion of physiologically grounded mechanisms related to synaptic scaling of local excitatory and inhibitory synapses, possibly in combination with the regulation of intrinsic excitability, their ability to recover pre-lesion properties and, more importantly, the local changes happening in parallel to this recovery of global function.

## Summary and Future Directions

Stroke is one of the most burdening diseases today in economically advantaged countries, not only by being the second largest cause of mortality but through the range of side-effects felt by stroke patients and their impact in life post-stroke (Campbell and Khatri, 2020). Therefore, it is essential to understand the changes operated in the brain that are direct effects of stroke lesions and the neural mechanisms of recovery that can restore function. Excitatory-inhibitory balance is one of the characteristics of neural activity essential to maintain several relevant functions, from sensory to higher-order association cortices (Wehr and Zador, 2003; Vogels et al., 2011; Xue et al., 2014; Hellyer et al., 2016). Here, we reviewed some of the relevant findings regarding EI balance and how it is maintained by homeostatic mechanisms in the face of external perturbations. Plastic processes known as synaptic scaling, operating in slow timescales, are ubiquitous and shown to contribute greatly to the maintenance of balance in cortical networks (Turrigiano et al., 1998; Hengen et al., 2013; Ma et al., 2019). In the context of stroke, due to the sudden loss of excitation from lesioned areas to connected regions of the cortex, it is reasonable to expect that such homeostatic process would play an important role in recovery. Indeed, the longitudinal variation of excitability in the cortex (Huynh et al., 2016) and, in particular, changes in the availability of excitatory (Qü et al., 1999; Jolkkonen et al., 2003) and inhibitory receptors (Jolkkonen et al., 2003; Kim et al., 2014), often in areas distant from the lesion, seem to point in that direction. Therefore, future attention should be devoted to investigating possible impairments in mechanisms such as synaptic scaling in stroke patients that could prevent cortical areas from compensating for lesion related imbalances. The therapeutic value of neuromodulation techniques aimed at increasing excitability is already known. Procedures such as tDCS and TMS (Blicher et al., 2009; Romero Lauro et al., 2014) have been applied in the past years, particularly targeted at modulation of motor cortex excitability, and future studies should focus on extending such therapies to different areas in the brain that might experience greater changes in normal levels of incoming excitation, possibly even in a patient-specific manner. Critically, it is important to stress that particular changes in excitability that can aid in the functional recovery of stroke patients might also have side effects. Indeed, increases in motor cortex excitability that are beneficial for motor recovery concurrently raise the risk of developing epileptic seizures (Jaenisch et al., 2016), a common side effect of stroke (Feyissa et al., 2019). Further symptoms of stroke such as depression (Concerto et al., 2013; Towfighi et al., 2017; Page and Coutellier, 2019), chronic pain (Klit et al., 2011; Ratté and Prescott, 2016) and fatigue (Kuppuswamy et al., 2015) have also been related to changes in the excitability of particular brain areas. Therefore, it is not out of the question that, in the process of compensating for diaschisis, homeostatic plasticity mechanisms lead to changes that might increase the propensity of stroke patients to develop the mentioned pathologies.

Another important aspect that is strongly affected by stroke is cortical functional connectivity. Either through changes in the strength of connections between particular areas (He et al., 2007; Carter et al., 2009; Corbetta et al., 2018) or more complex changes in the graph organization of functional networks (Wang et al., 2014; Siegel et al., 2016), stroke can cause deep disruptions in the function of large-scale cortical networks, spreading beyond lesioned areas. These are particularly related to decreased homotopic interhemispheric connectivity (Corbetta et al., 2018), increased segregation of functional networks and decreased modularity (Siegel et al., 2018), and can be, to some extent, returned to pre-lesion levels by spontaneous recovery. Indeed, from the evidence presented in the clinical, experimental and modeling studies discussed so far, it seems likely that the recovery of FC properties during stroke recovery is, to a certain level, dependent on the regulation of excitability at a local level. Previous research indicates that neuromodulation methods that target excitability of particular brain areas also lead to changes in connectivity (Nettekoven et al., 2014, 2015; Volz et al., 2015), suggesting a possibility of simultaneously manipulating cortical activity in both a local (excitability) and global (connectivity) scale. In consequence, given the unclear way both processes influence each other (Nettekoven et al., 2014), future studies must address the relationship between the maintenance of EI balance after stroke and the reorganization of functional networks, elucidating on a possible causal interaction between these two phenomena.

As previously mentioned, computational modeling can be of great use in this context by providing a more controllable and manipulable framework to study neurological disorders. More particularly, in the case of stroke, large-scale computational models of cortical activity would then allow the study of simultaneous changes in excitability and functional connectivity. More importantly, personalized stroke models could be applied to detect areas of particular impairment in a patient-specific manner, being further used to test the effects of therapies such as tDCS before they are applied in the clinical context. To address this, emphasis should be put on developing interactive modeling and visualization platforms (Arsiwalla et al., 2015) that allow the exploration of the dynamic evolution of cortical activity in stroke patients, aiding in the detection of specific impairments and targets for stimulation, including modeling in the pipeline of clinical decision making. Particular care should be given as well to the possibility of side effects arising from changes in excitability, which might be necessary to recover properties of FC networks, important for the performance of higher-order integrated functions such as memory, language and attention. Research should therefore focus on investigating the likelihood of developing impairments related to excitability in the process of restoring FC by correcting EI imbalances. Another approach would be to investigate the possibility of restoring FC without significant changes in excitability. However, in this case, particular areas of the cortex would require targeted manipulation. For example, while functional connectivity has been proved essential for associative functions (Goldman-Rakic, 1988; Mesulam, 1990), the recovery of visual and motor functions may not share such relation to the connectivity of visual and motor areas, being more dependent on the function these more peripheral cortices, which could suffer more if EI balance is not restored.

In conclusion, while connecting the local and global scales of diaschisis and stroke recovery is poised to be one of the main challenges in the future of stroke research, the study of EI balance and functional connectivity might be the direction to take toward a better understanding of these two aspects of cortical activity. Thus, shedding light into their interaction can be the key to understanding the range of side-effects of stroke and to designing and developing more efficient therapies, thus improving rehabilitation procedures for stroke patients. In addition, the localized nature of stroke lesions provides a framework to advance our understanding of cortical function, both at regional and network levels, clarifying how behavioral and cognitive functions emerge from the local and global properties of cortical activity.

## Author Contributions

FP and PFMJV contributed to the format and writing of the review and approved the final submitted version. FP contributed to the design and selection of the figures. Both authors contributed to the article and approved the submitted version.

## Conflict of Interest

FP is employed by company Eodyne Systems SL. PFMJV is founder and shareholder of Eodyne Systems S.L., which aims at bringing scientifically validated neurorehabilitation and education technology to society.

## Publisher’s Note

All claims expressed in this article are solely those of the authors and do not necessarily represent those of their affiliated organizations, or those of the publisher, the editors and the reviewers. Any product that may be evaluated in this article, or claim that may be made by its manufacturer, is not guaranteed or endorsed by the publisher.
